# Supplementary material for: MetaRibo-Seq measures translation in microbiomes
Source: Nat Commun. 2020 Jun 29;11:3268. doi: 10.1038/s41467-020-17081-z (PMC7324362; doi:10.1038/s41467-020-17081-z)
Supplement: Supplementary file 10 — Supplementary Data 7 [file 41467_2020_17081_MOESM10_ESM.zip › File2/Confidence_High_Taxonomy/225333_out.krona.html]

Javascript must be enabled to view this page.

members
magnitude
magnitudeUnassigned
count
unassigned
taxon
rank

225333\_out

74

2
superkingdom
74

1
1239
phylum

1
class
91061

1
order
1385

1
1378
genus


SRS019022\_contig\_number\_17375
84135
species
1

73
32066
phylum

73
203490
class

73
order
203491

family
203492
73

73
848
genus

71

SRS011310\_contig\_number\_883SRS013818\_contig\_number\_10SRS015038\_contig\_number\_contig-100\_11479.46501SRS015440\_contig\_number\_contig-100\_11879.75725SRS015592\_contig\_number\_contig-100\_1961.1962SRS015644\_contig\_number\_12847SRS015893\_contig\_number\_997SRS016342\_contig\_number\_4595SRS016653\_contig\_number\_contig-100\_5937.5938SRS017013\_contig\_number\_2604SRS017810\_contig\_number\_contig-100\_8176.42397SRS018055\_contig\_number\_contig-100\_17113.52917SRS018329\_contig\_number\_contig-100\_6211.28352SRS018610\_contig\_number\_contig-100\_8084.78695SRS018969\_contig\_number\_contig-100\_5744.117755SRS019587\_contig\_number\_contig-100\_5786.5786SRS019974\_contig\_number\_12908SRS019980\_contig\_number\_93SRS021496\_contig\_number\_contig-100\_413.413.413SRS021986\_contig\_number\_contig-100\_5544.5544SRS022079\_contig\_number\_contig-100\_5753.5753SRS022143\_contig\_number\_157SRS022625\_contig\_number\_contig-100\_8160.64338SRS023926\_contig\_number\_1692SRS023958\_contig\_number\_contig-100\_15803.49721SRS024347\_contig\_number\_contig-100\_9948.40151SRS024441\_contig\_number\_14131SRS043663\_contig\_number\_contig-100\_8608.154735SRS044486\_contig\_number\_562SRS047210\_contig\_number\_contig-100\_13461.77989SRS049253\_contig\_number\_contig-100\_21102.21102SRS051786\_contig\_number\_contig-100\_721.721SRS052518\_contig\_number\_contig-100\_12608.38915SRS053584\_contig\_number\_contig-100\_9275.61355SRS057389\_contig\_number\_contig-100\_8410.8411SRS063193\_contig\_number\_contig-100\_13158.143317SRS063373\_contig\_number\_contig-100\_8020.104091SRS064022\_contig\_number\_contig-100\_5666.46416SRS064617\_contig\_number\_contig-100\_157.158SRS074678\_contig\_number\_contig-100\_9229.64920SRS075404\_contig\_number\_9SRS075957\_contig\_number\_contig-100\_152.152SRS078671\_contig\_number\_contig-100\_11115.245936SRS097972\_contig\_number\_contig-100\_25837.96175SRS101384\_contig\_number\_289SRS101439\_contig\_number\_contig-100\_12568.130888SRS104087\_contig\_number\_contig-100\_14502.84203SRS104279\_contig\_number\_31958SRS104314\_contig\_number\_contig-100\_18846.58679SRS104384\_contig\_number\_1368SRS104704\_contig\_number\_10SRS104706\_contig\_number\_935SRS104830\_contig\_number\_21487SRS104964\_contig\_number\_contig-100\_225.226SRS1055028\_contig\_number\_23SRS1055060\_contig\_number\_contig-100\_38238.300702SRS142660\_contig\_number\_contig-100\_11890.11890SRS142896\_contig\_number\_24000SRS143135\_contig\_number\_contig-100\_11642.72715SRS143290\_contig\_number\_contig-100\_6415.6415SRS143310\_contig\_number\_8552SRS143678\_contig\_number\_contig-100\_12756.67856SRS143726\_contig\_number\_24515SRS146878\_contig\_number\_contig-100\_13753.100189SRS147664\_contig\_number\_142SRS148150\_contig\_number\_1475SRS148303\_contig\_number\_2151SRS148363\_contig\_number\_contig-100\_8022.36806SRS148771\_contig\_number\_contig-100\_12488.45656SRS893297\_contig\_number\_contig-100\_6516.208369SRS893363\_contig\_number\_26
species
860

species
1
851

SRS146750\_contig\_number\_contig-100\_16031.49715
2

1
76857
subspecies

SRS074976\_contig\_number\_contig-100\_21735.180437
